# *Notes from the Field:* Spatially Associated Coincident and Noncoincident Cases of La Crosse Encephalitis — North Carolina, 2002–2017

**DOI:** 10.15585/mmwr.mm6739a8

**Published:** 2018-10-05

**Authors:** Brian D. Byrd, Carl J. Williams, J. Erin Staples, Kristen L. Burkhalter, Harry M. Savage, Michael S. Doyle

**Affiliations:** ^1^Environmental Health Sciences Program, Western Carolina University, Cullowhee, North Carolina, ^2^North Carolina Division of Public Health, Raleigh, North Carolina, ^3^Division of Vector-Borne Diseases, National Center for Emerging and Zoonotic Infectious Diseases, CDC.

La Crosse virus (LACV) is the most common cause of pediatric arthropod-borne viral (arboviral) encephalitis in the United States (*1*). It is a California serogroup bunyavirus primarily transmitted by the eastern tree-hole mosquito (*Aedes triseriatus*) (*2*). LACV encephalitis is a reportable condition in North Carolina and is a nationally notifiable disease. In North Carolina, LACV encephalitis is the most common endemic arboviral disease reported in humans, with seven western counties accounting for approximately 80% of confirmed cases since 2003 (*3*). The fatality rate for LACV encephalitis is <1%, with most patients recovering without overt clinical sequelae; however, long-term neurologic sequelae reported in some patients include recurrent seizures, hemiparesis, and cognitive and neurobehavioral abnormalities (*4*).

In August 2017, the North Carolina Department of Public Health (NCDPH) was notified of a suspected LACV encephalitis case in a boy aged 2 years from western North Carolina. The following day, NCDPH was notified that the patient’s brother, aged 11 years, was also ill with symptoms consistent with viral meningoencephalitis. Laboratory testing confirmed that both siblings had evidence of recent LACV infection (Table).

**TABLE:** Coincident or spatially associated noncoincident La Crosse virus neuroinvasive disease cases — North Carolina, 2002–2017

Year (onset week)	Age, yrs (sex)	Association (residence)*	Laboratory evidence^†^	Outcome
Coincident cases				
2017 (30/31)	2 (M)	Sibling pair, same residence (A)	LACV IgM ELISA positive (CSF and serum)	Survived
	11 (M)		LACV IgM ELISA and PRNT positive (serum)	Survived
2011 (34)	5 (M)	Sibling pair, same residence (B)	LACV IgM ELISA and PRNT positive (CSF and serum)	Survived
	8 (F)		LACV IgM ELISA and PRNT positive (serum)	Died
			LACV RT-PCR positive (CSF)	
2010 (37)	4 (M)	Sibling pair, same residence (C)	LACV IgM ELISA positive (CSF and serum)	Survived
	6 (F)		LACV IgM ELISA positive (CSF and serum)	Survived
2002 (25/26)	8 (F)	Caregiver and child, same residence (D)	LACV IgM and IgG IFA positive (serum)	Survived
	32 (F)		LACV IgM and IgG IFA positive (serum x 2)	Survived
**Spatially linked noncoincident cases**				
2015 (29)	8 (F)	Sibling pair (E)	LACV IgM ELISA and PRNT positive (serum)	Survived
2011 (36)	6 (M)		LACV IgM ELISA positive (CSF and serum)	Survived
2012 (27)	4 (M)	No family relationship, home ownership changed (F)	LACV IgM ELISA positive (CSF and serum)	Survived
2005 (37)	5 (M)		LACV IgM ELISA positive (CSF and serum)	Survived
2011 (27)	6 (M)	No family relationship, linked to 2010 cases (same multi-building cluster) (C)	LACV IgM ELISA positive (CSF)	Survived

**Abbreviations:** CSF = cerebrospinal fluid; ELISA = enzyme linked immunosorbent assay; F = female; IFA = immunofluorescent assay; IgG = immunoglobulin G; IgM = immunoglobulin M; LACV = La Crosse virus; M = male; PRNT = plaque reduction neutralization test; RT-PCR = reverse transcription–polymerase chain reaction.

* Letters A–F indicate unique residences.

^†^ Testing performed at North Carolina Department of Public Health Public Health Laboratory and CDC Arbovirus Diagnostic Laboratory.

An interagency environmental assessment team who visited the siblings’ residence identified multiple risk factors associated with increased risk for LACV transmission (*5*). Water-filled artificial containers containing *Aedes* mosquito larvae (*Ae. triseriatus*, *Ae. albopictus*, and *Ae. japonicus*) were found on the premises, multiple windows and doors lacked effective screens, and the yard was within close proximity (<50 m) to a mixed hardwood forest. Additional mosquito samples (adult mosquitoes collected by large-bore aspirator and mosquitoes reared from eggs collected by ovitraps) were obtained; mosquitoes from egg collections were tested for LACV infection by real-time reverse transcription–polymerase chain reaction (RT-PCR) (*6*) and adult mosquitoes were tested by virus isolation attempts in cell culture; no virus was detected (*7*). The sibling pair was known to play outdoors daily and received mosquito bites during the expected incubation period (5–15 days) before disease onset.

To identify additional LACV patients among two or more persons residing at the same location, 331 confirmed or probable LACV case reports meeting the case definition* were reviewed in the North Carolina Electronic Disease Surveillance System. Three additional coincident patient pairs (two sibling pairs and one caregiver/child pair), linked spatially by place of residence and occurring during the same or sequential epidemiologic weeks, were identified (Table). In addition, three instances of confirmed LACV encephalitis were identified in children residing at the same residence, but during different years (spatially linked noncoincident cases). In one instance, the patients had no familial relationship but were linked by residence (residence F) after a change in home ownership. Three additional cases (one coincident sibling pair and one noncoincident case) were linked by residence within the same multibuilding apartment cluster (residence C).

These identified cases indicate that LACV disease risk can occur coincidently or noncoincidentally in time in persons at the same physical residence and further support surveillance data indicating that the disease is highly focal, occurring in a limited geographic area (*8*). This finding suggests that environmental assessments and modifications (e.g., filling tree holes, installing and repairing window or door screens, and removing water-filled containers) at locations where cases occur could help reduce the risk for this disease. In addition, persons living at the residence of a patient with a newly identified case of LACV disease or in an area where the virus is known to occur should be advised of the risk and measures to reduce risk, such as using Environmental Protection Agency–registered and recommended insect repellents, reducing time outdoors when mosquitoes are active, appropriate environmental modifications, and wearing clothing that prevents mosquito bites. Finally, health care providers should be aware of the potential clustering of LACV disease at a specific location and routinely advise their patients about mosquito prevention measures they can take to lower their risk. Additional information about LACV is available at https://www.cdc.gov/lac/index.html.

## Footnotes

* https://wwwn.cdc.gov/nndss/conditions/arboviral-diseases-neuroinvasive-and-non-neuroinvasive/.
